# SMC-specific IGF1 suppresses a 45 kDa LAMP2 isoform to mediate the anti-atherosclerotic effect of ferulic acid

**DOI:** 10.3389/fphar.2026.1849904

**Published:** 2026-08-06

**Authors:** Wentong Wu, Zhaoce Zheng, Danping Wang, Ming Shan, Jing Han, Dongqing Jing, Chunjng Liang, Xi Zhang, Jinxin Liu

**Affiliations:** 1 Clinical Experimental Research Center, Affiliated Hospital of Shandong Second Medical University (Clinical Medical School), Weifang, Shandong, China; 2 Geriatric Department, Affiliated Hospital of Shandong Second Medical University (Clinical Medical School), Weifang, Shandong, China; 3 College of Traditional Chinese Medicine, Shandong Second Medical University, Weifang, Shandong, China; 4 Department of Neurology, Affiliated Hospital of Shandong Second Medical University (Clinical Medical School), Weifang, Shandong, China; 5 School of Medical Imaging, Shandong Second Medical University, Weifang, Shandong, China; 6 General Practice Department, Affiliated Hospital of Shandong Second Medical University (Clinical Medical School), Weifang, Shandong, China

**Keywords:** atherosclerosis, ferulic acid, insulin-like growth factor-1, LAMP2, vascular smooth muscle cell

## Abstract

**Background and Aims:**

Ferulic acid (FA) exerts anti-atherosclerotic effects, but the underlying mechanisms remain incompletely understood. The present study investigated whether smooth muscle cell (SMC)-derived insulin-like growth factor 1 (IGF1) mediate FA’s protective effects by regulating lysosome-associated membrane protein 2 (LAMP2), inflammatory cytokines, and matrix metalloproteinase 3 (MMP3).

**Methods:**

Male ApoE^−/−^ mice were fed a high-fat diet for 8 weeks to established atherosclerosis models and then treated with FA (40 mg/kg/d). SMC-specific *Igf1* knockout (SMC-*Igf1*-KO) mice were generated to determine cell-type-specific requirements. *In vitro*, SMC-derived foam cells were induced by oxidized low-density lipoprotein (ox-LDL) and treated with FA ± IGF1 receptor (IGF1R) inhibitor. Plaque area, lipid deposition, and expression of IGF1, LAMP2, interleukin (IL)-6, IL-4, and MMP3 were assessed by *en face* staining, histology, Western blot, quantitative real-time PCR (qRT-PCR), and immunofluorescence selected as needed.

**Results:**

FA treatment significantly reduced atherosclerotic plaque area and lipid deposition in male ApoE^−/−^ mice, accompanied by upregulated IGF1 and downregulated 45 kDa-LAMP2 expression in aortic lesions. FA also increased IL-4 expression and decreased IL-6 and MMP3 expression. SMC-IGF1-KO significantly weakened the protective effects of FA. *In vitro*, FA increased IGF1 expression and IGF1R phosphorylation, reduced 45 kDa-LAMP2, IL-6, and MMP3 expression, and increased IL-4 expression in ox-LDL-induced SMC-derived foam cells. These effects were reversed by IGF1R inhibition.

**Conclusion:**

FA treatment significantly attenuates atherosclerosis. It partly resulted from that SMC-derived IGF1-mediated suppression of a 45 kDa-LAMP2 isoform, along with modulation of inflammatory balance (IL-6/IL-4) and MMP3 expression. The SMC-IGF1/45 kDa-LAMP2 axis may represent a potential therapeutic target for atherosclerosis.

## 1 Introduction

Atherosclerosis (AS) is the pathological basis of various cardiovascular diseases and remains a leading cause of death worldwide (Mensah et al., 2023). The rupture of atherosclerotic vulnerable plaque (AS-VP) is the main pathological and imaging cause of major adverse cardiovascular events (MACEs) (Virmani et al., 2006; Jia et al., 2013). AS is a chronic inflammatory and metabolic disease characterized by lipid deposition, smooth muscle cell (SMC) proliferation, intimal thickening, and plaque formation (Gu et al., 2021). It is composed primarily of lipid deposits, including cholesterol crystals, SMCs, and fibrous tissue, and progressively enlarges to cause luminal narrowing and reduced blood flow (Liu et al., 2023). In recent years, prevention and treatment strategies have evolved from passive management of thrombi and vascular occlusion to early intervention targeting vulnerable plaques (Gaba et al., 2023).

The lipid-rich necrotic core of AS-VP is mainly formed by foam cell aggregation and represents a hallmark of unstable lesions. Foam cells derive not only from monocyte-macrophages but also from SMCs (Allahverdian et al., 2014; Bartels et al., 2015; Kloc et al., 2022). Emerging evidence indicates that SMCs constitute a much larger proportion of plaque cells than previously estimated (Grootaert and Bennett, 2021), and they play a central role in the initiation and progression of AS (Bennett et al., 2016). Suppressing the transition of SMCs into foam cells may represent a key strategy for preventing or delaying AS (Engelen et al., 2022).

Our previous study demonstrated that decreased insulin-like growth factor 1 (IGF1) levels are independently correlated with the morphology of AS-VP, and IGF1 was inversely correlated with lumen stenosis and the lipid size in AS-VP (Liu et al., 2020). However, the role of IGF1 and its receptor pathway in the functional and phenotypic transformation of SMCs within AS-VP remains unclear.

Ferulic acid (FA) is a naturally occurring phenolic compound widely found in plants and has been reported to exert anti-atherosclerotic effect (Li et al., 2021a). FA may upregulate IGF1 (Tan et al., 2017) and modulate insulin/IGF1 signaling pathway (Li et al., 2021b). FA also inhibits the proliferation of SMCs (Wu et al., 2022), but the molecular mechanisms underlying its anti-atherosclerotic effects remain to be elucidated. Thus, we performed the present study to explore the anti-atherosclerotic effects and potential molecular mechanisms of FA, focusing on SMC differentiation and the IGF1/IGF1R pathway.

## 2 Materials and methods

### 2.1 Animals and treatments

All animal procedures were approved by the Institutional Animal Care and Use Committee of Shandong Second Medical University (Approval No. *2024SDL817*) and conformed to the National Institutes of Health Guide for the Care and Use of Laboratory Animals.

Male ApoE^−/−^ mice (8 weeks old, C57BL/6 J background) were purchased from Cyagen Biosciences (Suzhou, China). Mice were fed a high-fat, high-cholesterol diet (HFCD; 20% lard, 2% cholesterol, 0.2% choline bitartrate) for 8 weeks to induce atherosclerosis, then added FA treatment for a month. FA-treated mice received daily gavage of FA (40 mg/kg/d; Yuanye, China) dissolved in 0.25 mL corn oil (Aladdin Bio-Chem Technology Co., Ltd., China), while control groups received standard chow and daily gavage with autoclaved water. Totally 24 experimental mice were used in the group intervention experiment, with six mice per group, grouped as follows:

Control group: Standard chow + daily gavage with water in *ApoE* ^−/−^ mice.

Model group: HFCD + daily gavage with sterile corn oil in *ApoE* ^−/−^ mice.

FA group: HFCD + daily gavage with FA (40 mg/kg/d) dissolved in sterile corn oil in *ApoE* ^−/−^ mice.

FA + KO group: HFCD + FA (40 mg/kg/d) dissolved in sterile corn oil in [SMC-*Igf1*-KO][*ApoE*-KO] mice.

All gavages were administered at 10 μL/g body weight. Mice were sacrificed at 20 weeks of age for sample collection.

### 2.2 Generation of SMC-specific IGF1 knockout mice

SMC-specific *Igf1* knockout on *ApoE* ^−/−^ background was generated by intercrossing *ApoE* ^−/−^; *Igf1* ^flox/flox^ (Cyagen) and *ApoE* ^−/−^; *SMMHC*-Cre^ERT2^ (Cyagen, Y-linked) lines. The *SMMHC*-Cre^ERT2^ transgene expresses Cre recombinase under the smooth muscle-specific myosin heavy chain promoter. Genotyping was confirmed by PCR from tail DNA. Male *ApoE* ^−/−^; *Igf1* ^flox/flox^; *SMMHC*-Cre^ERT2^ (SMC-*Igf1*-KO) mice were used for atherosclerosis studies.

### 2.3 Quantification of atherosclerosis

Mice were euthanized using CO2 at a 60% flow rate of chamber volume per minute after 8 weeks of HFCD feeding. Hearts and aortas were perfused with cold 0.9% saline followed by 4% paraformaldehyde. The aorta was dissected from the aortic root to the iliac bifurcation. The aorta from the aortic arch to the bifurcation of the first intercostal artery is opened longitudinally stained with Oil Red O (Solarbio, China) to quantify *en face* lesion area. And the remaining aorta was used for Western blot experiments. Totally six sections per mouse at 40 µm intervals through the aortic root were analyzed for all staining, and three aortic root sections per mouse were analyzed for H&E or Oil Red O staining respectively. For aortic root analysis, frozen sections (8 μm) were stained with Oil Red O and hematoxylin. Lesion area and lipid deposition were quantified using ImageJ or Image-Pro Plus software.

### 2.4 Immunofluorescence analysis

Serial 8 μm frozen aortic root sections were prepared from OCT-embedded samples. Sections were incubated with mouse anti-α-SMA (Proteintech, China) together with one of the following primary antibodies: rabbit anti-IGF1 (Novus Biologicals), anti-LAMP2 (Absin, China), or anti-MMP3 (Santa Cruz Biotechnology). Primary antibodies were incubated overnight at 4 °C.

Sections were then incubated with fluorescent secondary antibodies (Alexa Fluor™, ThermoFisher; or TRITC-IgG, ZSGB-Bio) for 1 h at room temperature in the dark. Images were captured using a Zeiss fluorescence microscope equipped with an Axiocam 208 camera. The percentage of positively stained area relative to the vascular wall area was quantified using Zen 3.3 software.

### 2.5 Western blotting

Total protein was extracted from mouse aortic tissues or SMC pellets using lysis buffer supplemented with PMSF and phosphatase inhibitors, followed by incubation on ice. Protein concentrations were measured using a BCA assay. Equal amounts of protein were resolved by SDS-PAGE and transferred to PVDF membranes (Millipore, Germany). After blocking with 3% BSA, membranes were incubated overnight at 4 °C with primary antibodies.

After washing, membranes were incubated with HRP-conjugated secondary antibodies for 1 h at room temperature. Signal detection was performed using chemiluminescence, and band intensities were quantified using ImageJ software.

### 2.6 Cell counting Kit-8 assay

A mouse SMC line (MVSMC) was kindly provided by the Key Laboratory of Myocardial Ischemia, Harbin Medical University. Cells were cultured in DMEM containing 10% FBS, 100 U/mL penicillin, and 100 μg/mL streptomycin at 37 °C in 5% CO_2_. Cells between passages 3–6 were used for experiments.

To determine the optimal FA concentration and its effect on cell viability, SMCs were treated with various concentrations of FA (0, 20, 40, 80, 160, 320 μM; Shanghai Biotechnology Co.) for 24 h. Cells were then incubated with 10 μL CCK-8 solution (Dojindo, Japan) at 37 °C for 2 h. Absorbance at 450 nm was measured to calculate cell viability.

### 2.7 Culture of SMC-derived foam cells

When cells reached 70%–80% confluence, SMC-derived foam cells were induced using ox-LDL (80 μg/mL; Yiyuan Biotechnology). A 500 mM stock solution was prepared by dissolving 97 mg of FA in 1 mL of DMSO (Solarbio, China) and serially diluted in culture medium to a final concentration of 40 μM.

Cells were divided into five groups:

Control: Serum-free medium for 24 h, then complete medium for 24 h.

Model: ox-LDL (80 μg/mL) for 24 h, then complete medium for 24 h.

DMSO: ox-LDL (80 μg/mL) for 24 h, then DMSO (10 μL) for 24 h.

FA: ox-LDL (80 μg/mL) for 24 h, then FA (40 μM) for 24 h.

PPP: ox-LDL (80 μg/mL) for 24 h, then FA (40 μM) + PPP (0.48 μM; Shyuanye, China) for 24 h.

### 2.8 Quantitative real-time PCR

The cDNA was synthesized using the First Strand cDNA Synthesis kit (AG Bio, China) and used for the 40-cycle 2-step PCR with sequence-specific primer pairs in the iCycler IQ Real-Time Detection System (Bio-Rad). Relative gene expression was calculated using the 2^−^ΔΔCt method and normalized to GAPDH. The primers were designed by Sangon Biotech (Shanghai) Co., Ltd (Shanghai, China) (Table 1).

**TABLE 1:** Primer sequences for quantitative real-time PCR.

Gene	Forward primer (5'→3′)	Reverse primer (5'→3′)
IGF1	GCT​CTG​CTT​GCT​CAC​CTT​CAC​C	CGG​TCC​ACA​CAC​GAA​CTG​AAG​AG
LAMP2	CCA​CTC​CAA​CTC​CAA​CTC​CAA​CTC	GGT​AGC​CAG​CAG​ACA​GGT​AGT​ATT​G
MMP3	GAC​GAT​GAT​GAA​CGA​TGG​ACA​GAG​G	TGT​GGA​GGA​CTT​GTA​GAC​TGG​GTA​C
GAPDH	GGC​AAA​TTC​AAC​GGC​ACA​GTC​AAG	TCG​CTC​CTG​GAA​GAT​GGT​GAT​GG

Primer sequences for quantitative real-time PCR., All primers were synthesized by Sangon Biotech (Shanghai, China). GAPDH, was used as the internal control. Aberreviations: IGF1, insulin-like growth factor 1; LAMP2, lysosome-associated membrane protein 2; MMP3, matrix metalloproteinase 3; GAPDH, glyceraldehyde-3-phosphate dehydrogenase.

### 2.9 Fasting blood glucose (FBG) and lipid profile tests

All the mice were kept fasting overnight prior to euthanasia. On the following morning, eyeballs were extracted and blood was collected for serum preparation. FBG levels were measured using a glucometer (Jiangsu Yuyue Medical Equipment & Supply Co., Ltd., China).

Moreover, the sample still exhibited severe chylemia even after high-speed centrifugation, resulting in the inability to perform lipid profile testing.

### 2.10 Statistical analysis

Data are presented as mean ± SD. Normality was assessed using the Shapiro-Wilk test, and homogeneity of variance was assessed using the Brown-Forsythe test. Group differences were analyzed using one-way ANOVA followed by Tukey’s *post hoc* test for multiple comparisons, or Student’s t-test for two-group comparisons. A two-tailed P < 0.05 was considered statistically significant. Statistical analyses were performed using GraphPad Prism (version 9.0) or SPSS (version 20.0).

## 3 Results

### 3.1 Generation and validation of SMC-specific *Igf1* knockout mice

**FIGURE 1:**
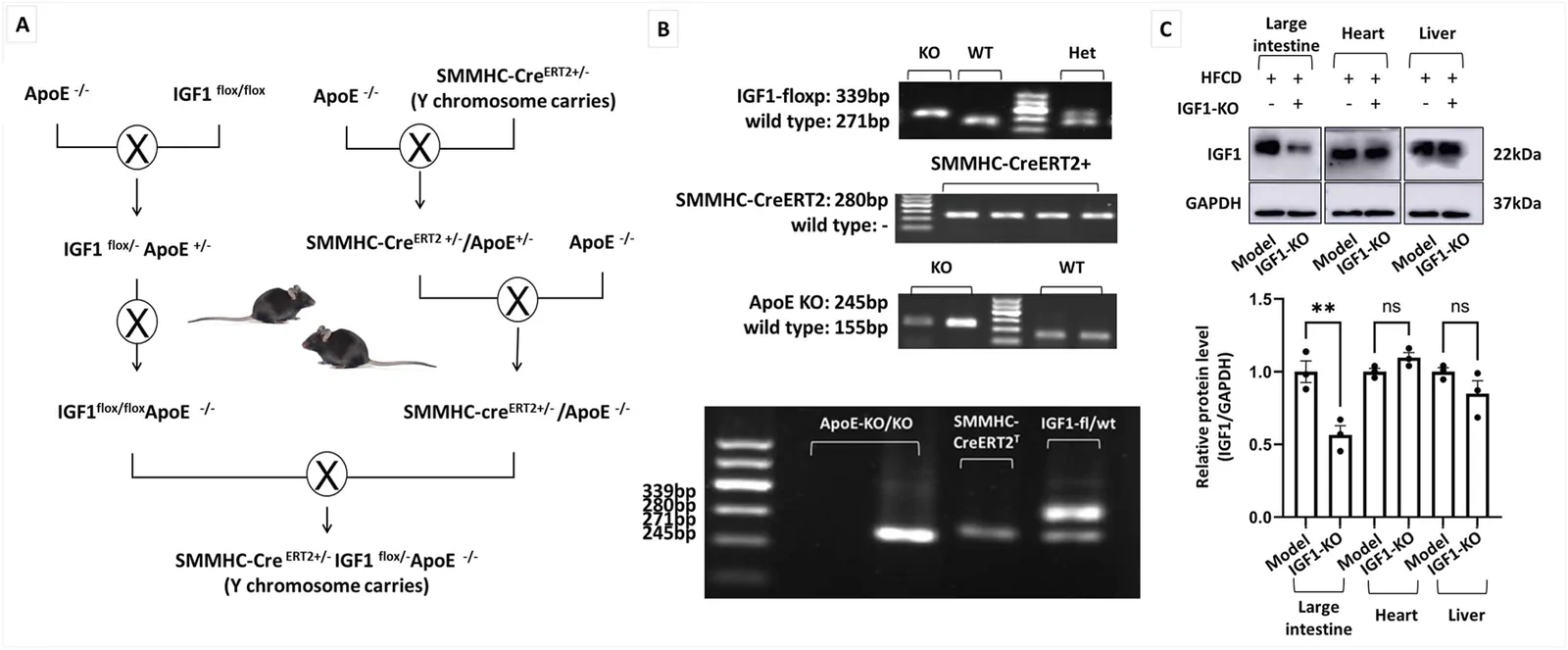
Generation and validation of SMC-specific IGF1 knockout mice. **(A)** Breeding strategy for generating SMC-IGF1-KO mice on an ApoE^−/−^ background. IGF1^(flox/flox); ApoE^−/−^ mice were crossed with SMMHC-CreERT2; ApoE^−/−^ mice. Tamoxifen induces SMC-specific IGF1 deletion. **(B)** Representative PCR genotyping showing band patterns for wild-type (WT), heterozygous (Het), and homozygous (KO) mice. **(C)** Validation of SMC-specific IGF1 knockout by Western blot analysis of IGF1 expression in colon, heart, and liver tissues. GAPDH served as loading control. IGF1 expression was significantly reduced in colon tissue of SMC-IGF1-KO mice, while no significant changes were observed in heart or liver tissues. N = 3 per group. Data are mean ± SD. **P < 0.01 vs. Control by Student’s t-test.

**FIGURE 2:**
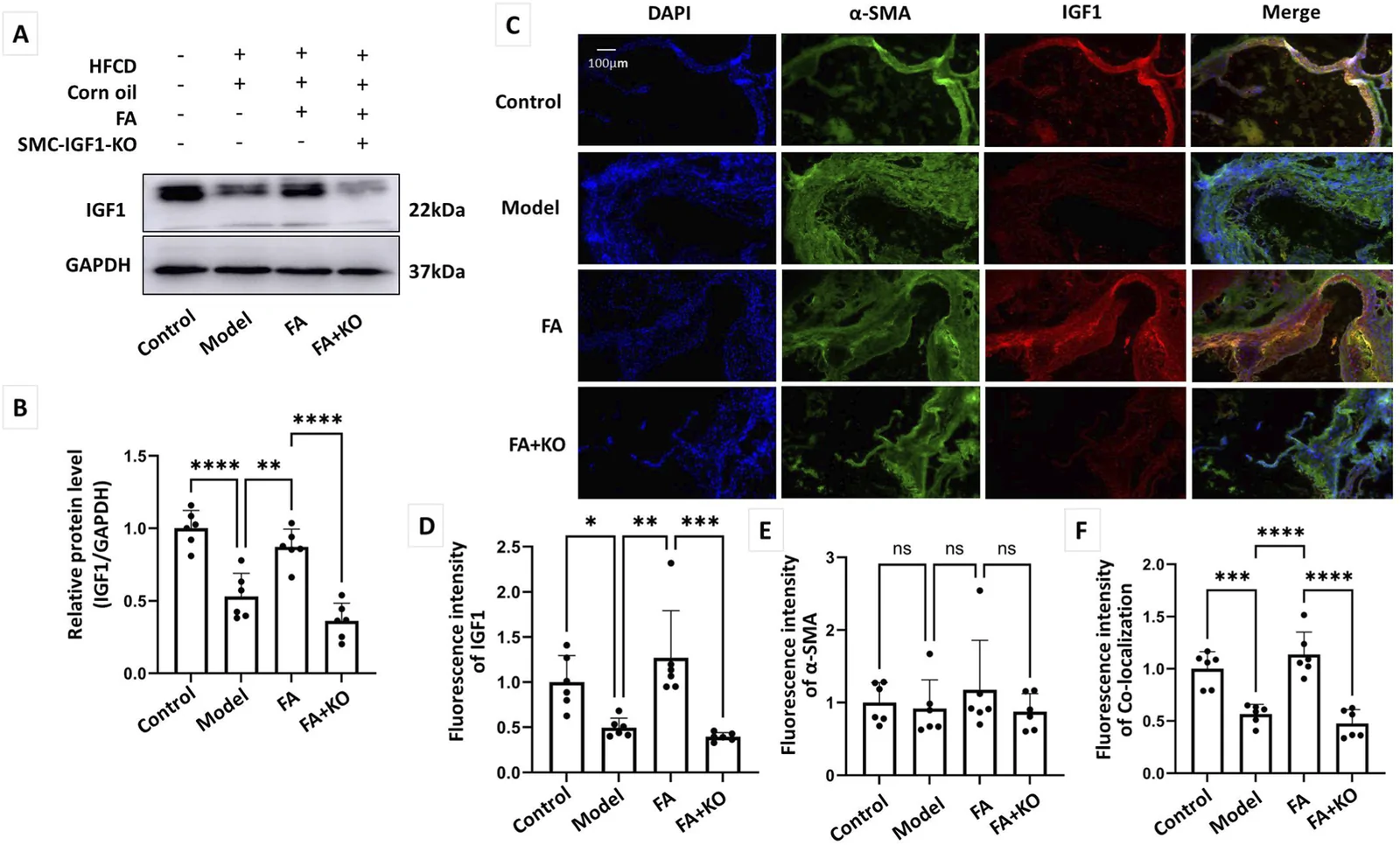
FA upregulates SMC-derived IGF1 expression. **(A)** Representative Western blots of IGF1 in aortic lysates. GAPDH served as loading control. **(B)** Densitometric quantification. **(C)** Representative immunofluorescence staining of IGF1 (green) and α-SMA (red) in aortic root sections. DAPI (blue) stains nuclei. Co-localization (yellow) indicates SMC-derived IGF1. Scale bar = 100 μm. **(D)** Comparison of IGF1 fluorescence intensity among groups. **(E)** Comparison of α-SMA fluorescence intensity among groups. **(F)** Comparison of IGF1/α-SMA co-localization fluorescence intensity among groups. N = 6 per group. Data are mean ± SD. **P < 0.01, ***P < 0.001 vs. indicated groups by one-way ANOVA with Tukey’s *post hoc* test.

Smooth muscle cell (SMC)-specific, tamoxifen-inducible *Igf1* conditional knockout mice on an *ApoE* ^−/−^ background were generated using Cre-loxP recombination (Figures 1A,B). Successful SMC-specific knockout was confirmed through two complementary approaches. First, Western blot analysis of IGF1 expression in colon, heart, and liver tissues showed that IGF1 levels were significantly reduced in the colon—a tissue rich in smooth muscle—of SMC-*Igf1*-KO mice compared with control mice, whereas no significant differences were observed in heart or liver tissues (Figure 1C). Second, functional validation in the context of atherosclerosis revealed that IGF1 expression in the aortic tissue of SMC-*Igf1*-KO mice was markedly reduced following ferulic acid treatment (Figures 2A,B), confirming efficient knockout under experimental conditions. Collectively, these findings demonstrate the successful generation of SMC-specific *Igf1* knockout mice.

### 3.2 FA attenuates atherosclerotic plaque formation in an IGF1-dependent manner

**FIGURE 3:**
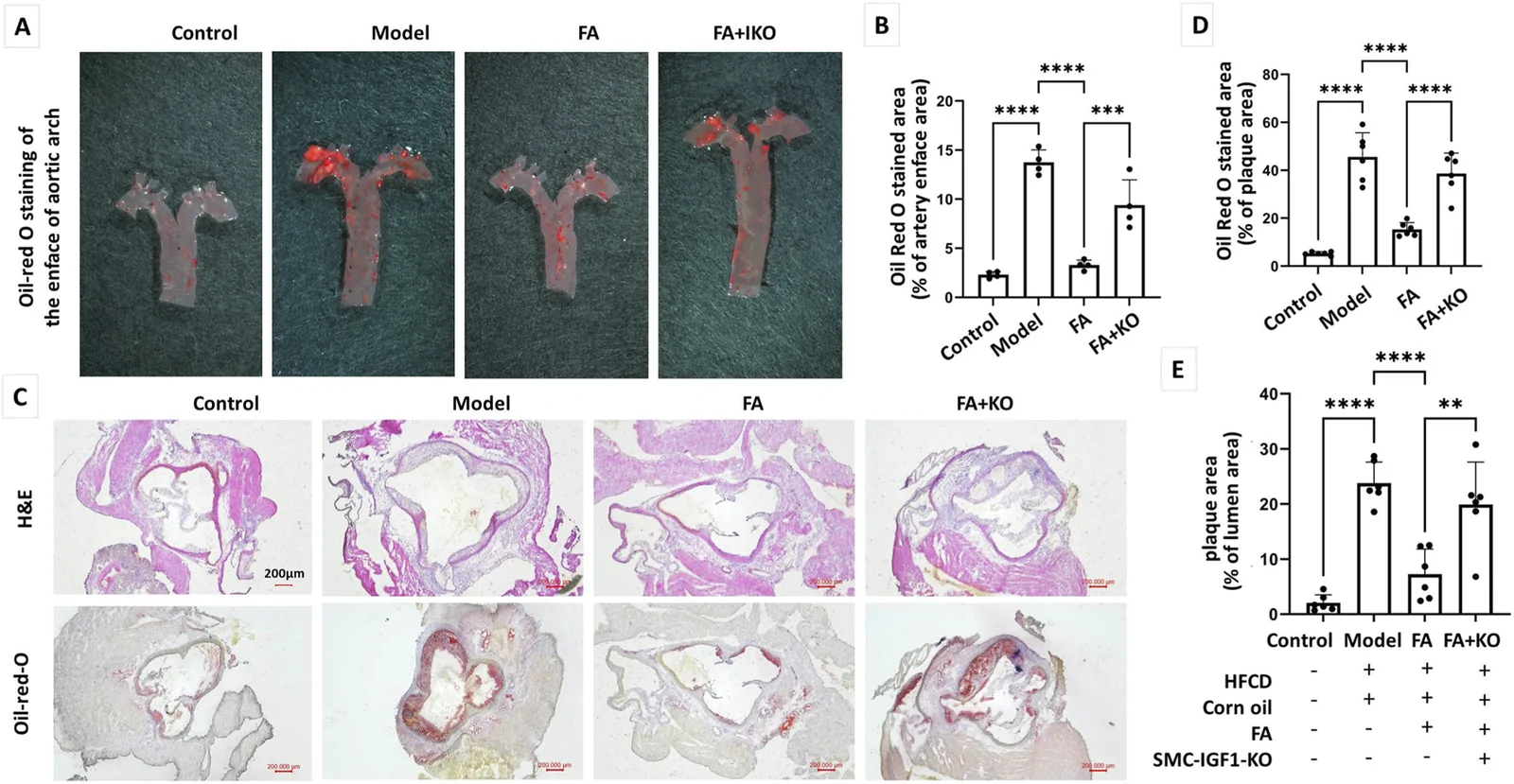
FA attenuates atherosclerotic plaque formation in an IGF1-dependent manner. **(A)** Representative *en face* Oil Red O staining of aortas. **(B)** Quantification of lesion area (Oil Red O). **(C)** Representative H&E (top) and Oil Red O (bottom) staining of aortic root sections. Scale bar = 200 μm. **(D)** Quantification of lipid deposition (Oil Red O). **(E)** Quantification of plaque area (H&E). N = 6 per group. Data are mean ± SD. *P < 0.05, **P < 0.01, ***P < 0.001, ****P < 0.0001 vs. indicated groups by one-way ANOVA with Tukey’s *post hoc* test.

*En face* Oil Red O staining of aortas (Figures 3A,B) and H&E and Oil Red O staining of aortic root sections (Figures 3C–E) were used to evaluate the impact of FA on plaque burden and composition. Compared with the control group, model mice exhibited markedly increased atherosclerotic plaque area and lipid content (both P < 0.0001). FA treatment significantly reduced plaque area and lipid accumulation compared with the model group.

Notably, the protective effects of FA were significantly attenuated in SMC-*Igf1*-KO mice. Compared with the FA group, FA-treated SMC-*Igf1*-KO mice showed significantly increased plaque area (P = 0.0013) and lipid content (P = 0.0004) (Figure 3). These findings indicate that FA inhibits atherosclerotic lesion development and that SMC-derived IGF1 is required for this protective effect.

### 3.3 FA upregulates SMC-derived IGF1 expression in atherosclerotic plaques

IGF1 protein expression in aortic tissue was assessed by Western blot (Figures 2A,B). Compared with the control group, model mice showed significantly reduced IGF1 levels (P = 0.0023). FA treatment markedly increased IGF1 expression compared with the model group (P < 0.0001) and restored IGF1 levels in SMC-*Igf1*-KO mice.

Immunofluorescence staining of aortic root sections revealed co-localization of IGF1 and α-SMA (Figures 2C–F). Co-staining analysis showed that FA significantly increased IGF1 expression and that in SMCs compared with the model group (P < 0.0001), and this effect was abolished in SMC-*Igf1*-KO mice. These findings suggest that FA primarily increases IGF1 expression in SMCs within atherosclerotic lesions.

### 3.4 FA modulates inflammatory cytokines and MMP3 expression in atherosclerotic plaques

**FIGURE 4:**
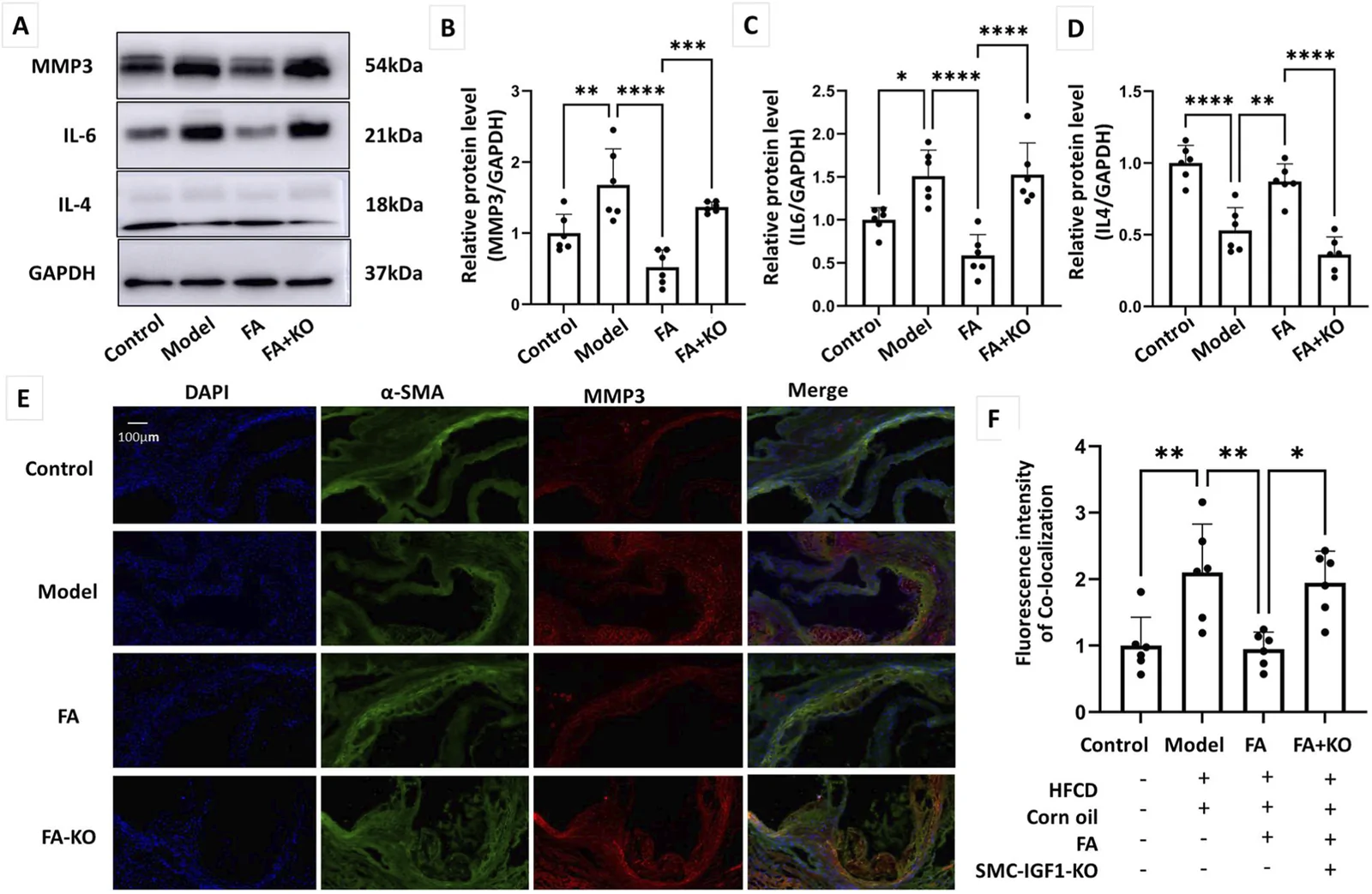
FA modulates inflammatory cytokines and MMP3 expression in atherosclerotic plaques. **(A)** Representative Western blots of MMP3, IL-6, and IL-4 in aortic lysates. GAPDH served as loading control. **(B–D)** Densitometric quantification of MMP3 **(B)**, IL-6 **(C)**, and IL-4 **(D)**. N = 6 per group. **(E)** Representative immunofluorescence staining of MMP3 (red) and α-SMA (green) in aortic root sections. DAPI (blue) stains nuclei. Co-localization (yellow) indicates SMC-derived MMP3. Scale bar = 100 μm. **(F)** Quantification of MMP3-positive area relative to α-SMA-positive area. N = 6 per group. Data are mean ± SD. *P < 0.05, **P < 0.01, ***P < 0.001, ****P < 0.0001 vs. indicated groups by one-way ANOVA with Tukey’s *post hoc* test.

Western blot analysis revealed that model mice exhibited significantly elevated expression of the pro-inflammatory cytokine IL-6 (P = 0.0022) and matrix metalloproteinase MMP3 (P = 0.0050), along with decreased expression of the anti-inflammatory cytokine IL-4 (P = 0.0001), compared with control mice (Figures 4A–D). FA treatment significantly reduced IL-6 (P < 0.0001) and MMP3 (P < 0.0001) levels and increased IL-4 expression (P = 0.0022). These changes were reversed by SMC-specific *Igf1* deletion (Figures 4A–D).

Immunofluorescence staining confirmed that FA reduced MMP3 expression in SMCs, and this effect was abolished in SMC-IGF1-KO mice (Figures 4E,F). These results indicate that FA suppresses inflammation and extracellular matrix degradation through IGF1-dependent mechanisms.

### 3.5 FA suppresses 45 kDa LAMP2 expression in atherosclerotic plaques

**FIGURE 5:**
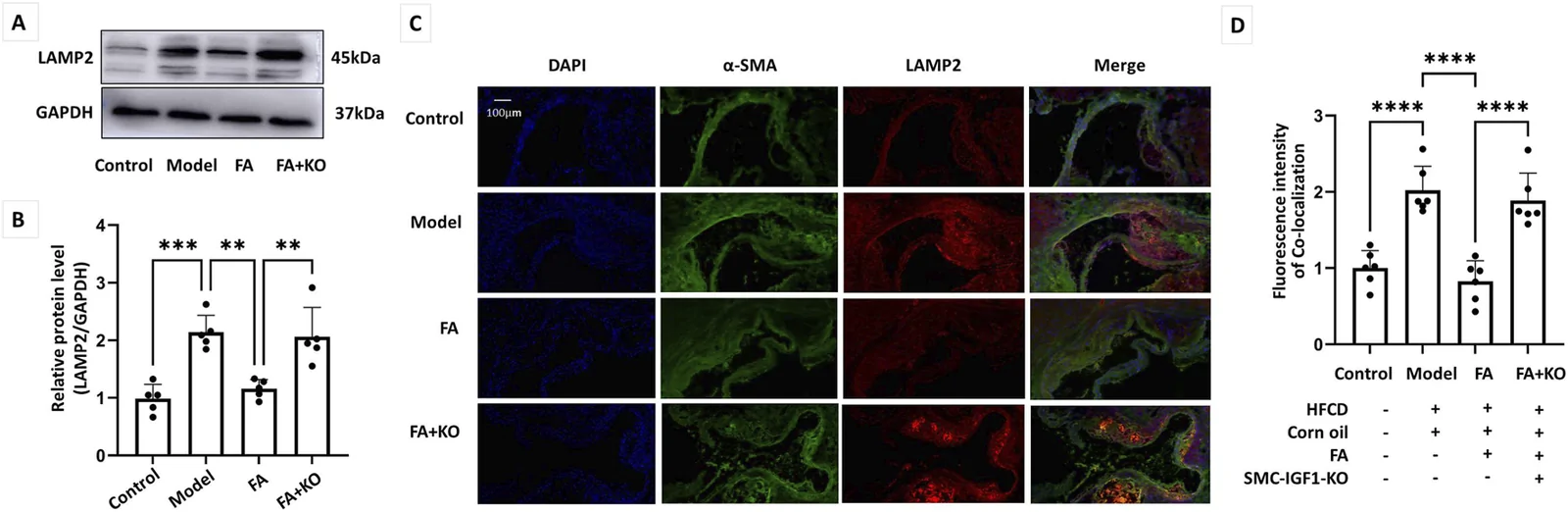
FA suppresses 45 kDa LAMP2 expression in SMCs. **(A)** Representative Western blots of LAMP2 in aortic lysates. GAPDH served as loading control. **(B)** Densitometric quantification. **(C)** Representative immunofluorescence staining of LAMP2 (red) and α-SMA (green) in aortic root sections. DAPI (blue) stains nuclei. Co-localization (yellow) indicates SMC-derived LAMP2. Scale bar = 100 μm. **(D)** Quantification of LAMP2-positive area relative to α-SMA-positive area. N = 6 per group. Data are mean ± SD. *P < 0.05 vs. indicated groups by one-way ANOVA with Tukey’s *post hoc* test.

LAMP2 expression in atherosclerotic plaques was assessed by Western blot (Figures 5A,B). Model mice showed significantly increased LAMP2 expression compared with control mice (P = 0.0140). FA treatment markedly reduced plaque LAMP2 levels (P = 0.0361), whereas SMC-specific *Igf1* deletion abolished this reduction (P = 0.0184).

Immunofluorescence co-staining of LAMP2 and α-SMA confirmed that FA reduced LAMP2 expression in SMCs, and this effect was reversed by SMC-specific *Igf1* deletion (Figures 5C,D). These results suggest that FA suppresses 45 kDa LAMP2 expression in SMCs through IGF1-related mechanisms.

### 3.6 FA activates IGF1R signaling in SMC-derived foam cells

**FIGURE 6:**
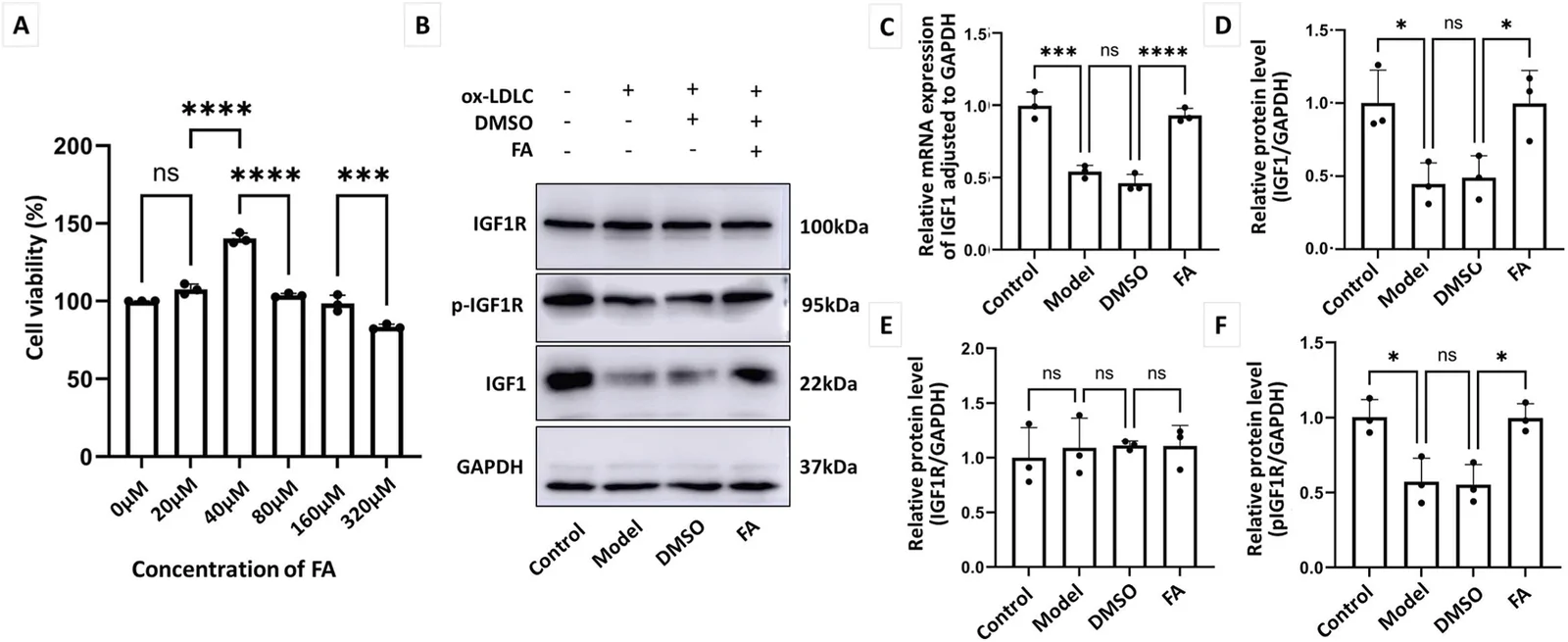
FA Activates IGF1R Signaling in SMC-Derived Foam Cells. **(A)** VSMC viability assessed by CCK-8 assay after 24 h treatment with indicated FA concentrations. N = 6 per group. **(B)** Representative Western blots of IGF1R, p-IGF1R, and IGF1 in SMCs treated as indicated. GAPDH served as loading control. **(C)** IGF1 mRNA expression by qRT-PCR, normalized to GAPDH. **(D–F)** Densitometric quantification of IGF1R **(D)**, p-IGF1R **(E)**, and IGF1 **(F)**. N = 3 independent experiments. Data are mean ± SD. *P < 0.05, ***P < 0.001, ****P < 0.0001 vs. indicated groups by one-way ANOVA with Tukey’s *post hoc* test.

CCK-8 assay showed that FA at concentrations up to 40 μM did not affect SMC viability (Figure 6A). Western blot and qRT-PCR analysis revealed that model SMCs exhibited significantly decreased expression of p-IGF1R (P = 0.0114) and IGF1 (P = 0.0296) compared with control cells (Figures 6B–F). FA treatment restored p-IGF1R (P = 0.0255) and IGF1 (P = 0.0353) levels. No significant differences were observed between the model and DMSO groups. IGF1R expression levels did not differ significantly among groups. These results indicate that FA activates the IGF1/IGF1R signaling pathway by upregulating IGF1 and increasing IGF1R phosphorylation rather than by altering IGF1R quantity.

### 3.7 FA modulates inflammation and MMP3 expression in SMC-derived foam cells

**FIGURE 7:**
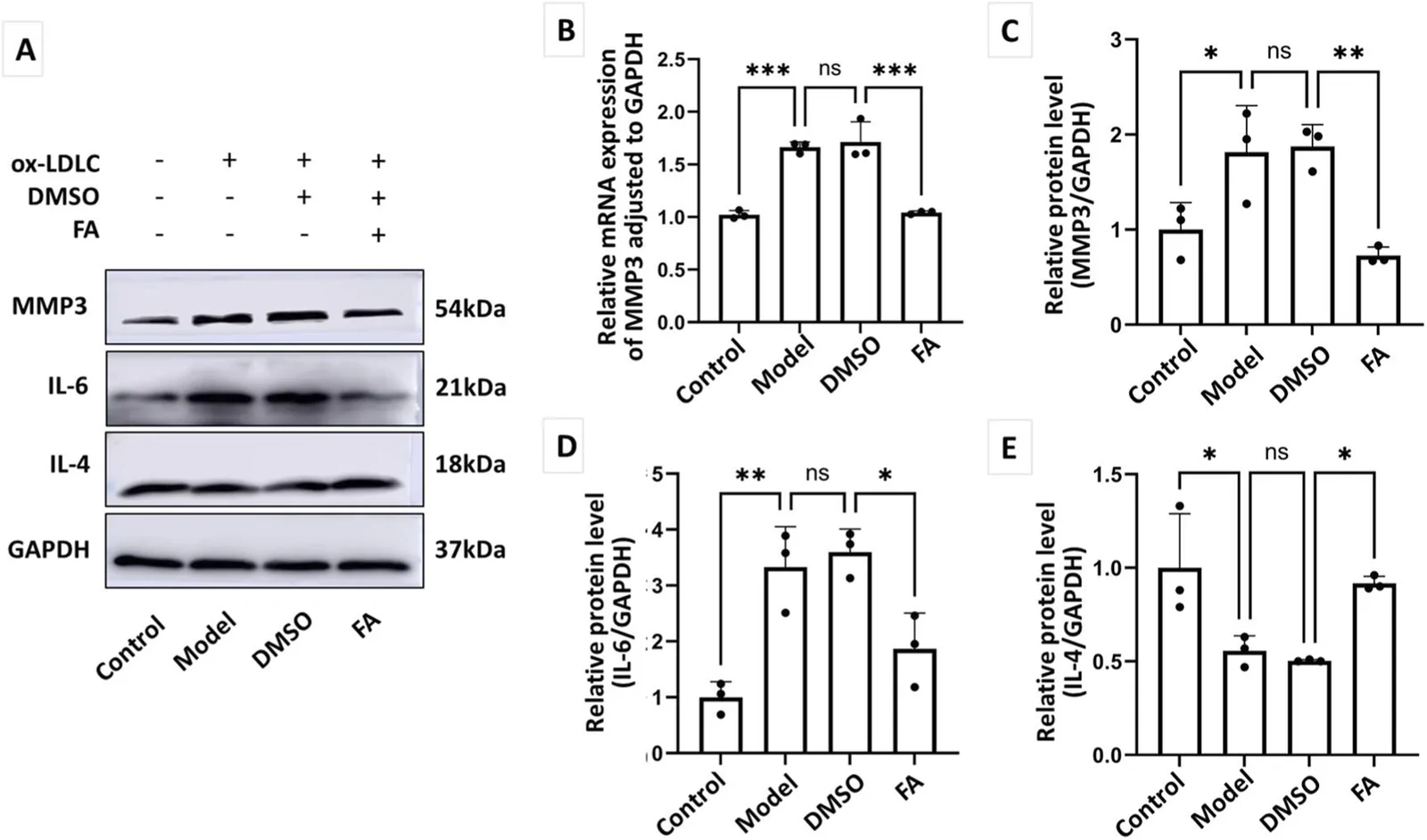
FA modulates inflammatory cytokines and MMP3 expression in SMC-derived foam cells. **(A)** Representative Western blots of MMP3, IL-6, and IL-4 in SMCs treated as indicated. GAPDH served as loading control. **(B)** MMP3 mRNA expression by qRT-PCR, normalized to GAPDH. **(C–E)** Densitometric quantification of MMP3 **(C)**, IL-6 **(D)**, and IL-4 **(E)**. N = 3 independent experiments. Data are mean ± SD. *P < 0.05, **P < 0.01, ***P < 0.001 vs. indicated groups by one-way ANOVA with Tukey’s *post hoc* test.

Western blot and qRT-PCR analysis showed that model SMCs exhibited significantly increased expression of IL-6 (P = 0.0212) and MMP3 (P = 0.0222) and decreased expression of IL-4 (P = 0.0205) compared with control cells (Figure 7). FA treatment significantly reduced IL-6 (P = 0.0477) and MMP3 (P = 0.0031) levels and increased IL-4 expression (P = 0.0459). No significant differences were observed between the model and DMSO groups. These results confirm that FA suppresses inflammation and extracellular matrix degradation in SMC-derived foam cells.

### 3.8 FA reduces LAMP2 expression in SMC-derived foam cells

**FIGURE 8:**
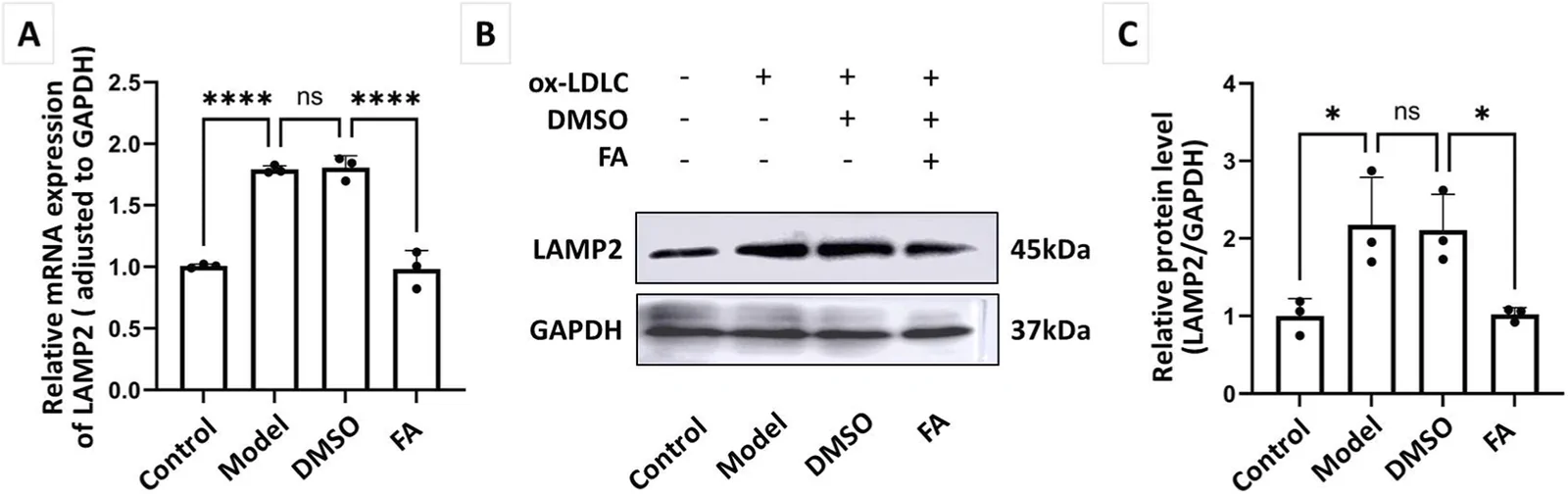
FA reduces LAMP2 expression in SMC-derived foam cells. **(A)** Representative Western blots of LAMP2 in SMCs treated as indicated. GAPDH served as loading control. **(B)** LAMP2 mRNA expression by qRT-PCR, normalized to GAPDH. **(C)** Densitometric quantification of LAMP2 protein levels. N = 3 independent experiments. Data are mean ± SD. *P < 0.05, ****P < 0.0001 vs. indicated groups by one-way ANOVA with Tukey’s *post hoc* test.

LAMP2 expression was assessed by Western blot and qRT-PCR (Figure 8). Model SMCs showed significantly increased LAMP2 expression compared with control cells (P = 0.0204 b y Western blot; P < 0.0001 b y qRT-PCR). FA treatment significantly reduced LAMP2 expression (P = 0.0491 b y Western blot; P < 0.0001 b y qRT-PCR), whereas no significant differences were observed between the model and DMSO groups. These results indicate that FA suppresses LAMP2 expression in SMC-derived foam cells.

### 3.9 IGF1/IGF1R inhibition abrogates FA’s protective effects

**FIGURE 9:**
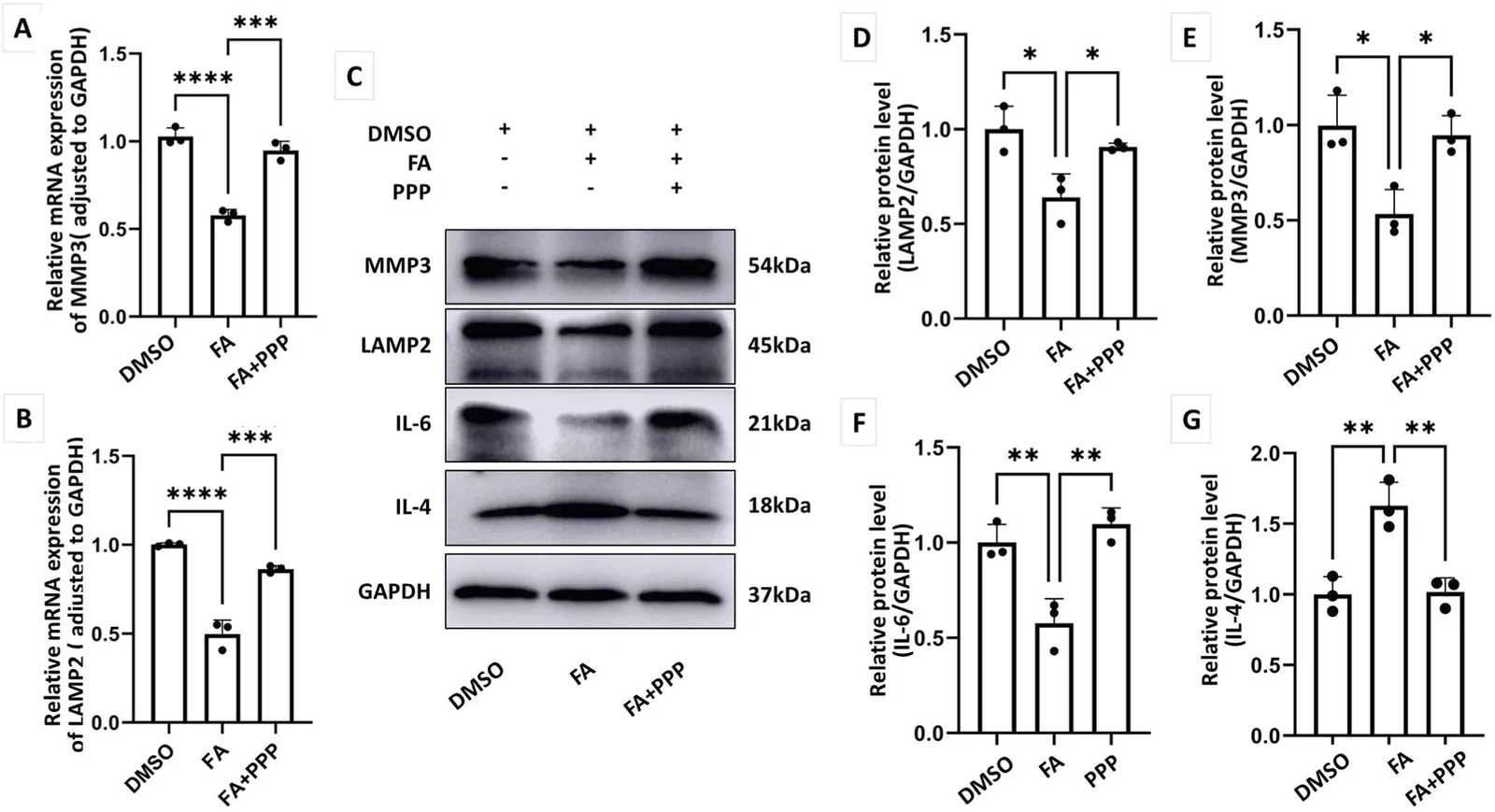
IGF1/IGF1R inhibition abrogates FA’s protective effects in SMC-derived foam cells. **(A,B)** MMP3 and LAMP2 mRNA expression by qRT-PCR, normalized to GAPDH. **(C)** Representative Western blots of MMP3, LAMP2, IL-6, and IL-4 in SMCs treated as indicated. GAPDH served as loading control. **(D–G)** Densitometric quantification of LAMP2 **(D)**, MMP3 **(E)**, IL-6 **(F)**, and IL-4 **(G)**. N = 3 independent experiments. Data are mean ± SD. *P < 0.05, **P < 0.01, ***P < 0.001, ****P < 0.0001 vs. indicated groups by one-way ANOVA with Tukey’s *post hoc* test.

The IGF1R inhibitor picropodophyllin (PPP) was used to investigate whether the IGF1/IGF1R pathway mediates the regulatory effects of FA on SMCs (Figure 9). Compared with DMSO controls, FA treatment significantly decreased IL-6 (P = 0.0173), MMP3 (P = 0.0279), and LAMP2 (P = 0.0011) expression and increased IL-4 expression (P = 0.0345). Co-treatment with PPP significantly reversed these FA-induced changes, increasing IL-6 (P = 0.0060), MMP3 (P = 0.0381), and LAMP2 (P = 0.0194) expression and decreasing IL-4 expression (P = 0.0424). These results indicate that inhibition of the IGF1/IGF1R pathway attenuates the protective effects of FA in SMC-derived foam cells.

### 3.10 FA had no significant effect on FBG levels in experimental mice

Fasting blood glucose levels were measured in all experimental groups. Two-way ANOVA revealed significant effects of HFCD feeding and SMC-specific *Igf1* deletion (p = 0.008) on blood glucose, while FA treatment showed no significant effect compared with Model group (p = 0.063). These data suggest that the atheroprotective effect of FA is not mediated through systemic glucose regulation (Table 2).

**TABLE 2:** Comparison of FBG test results between groups.

Group	Glucose (mmol/L)	P value
Control	5.033 ± 1.285	0.004^*^
Model	6.883 ± 1.748	0.083^#^
FA	4.983 ± 1.385	0.063^†^
FA + KO	7.833 ± 1.113	0.008^‡^

* the P-value for the four group comparisons.

# the P-value for comparing Group Model with Group Control.

† the P-value for comparing Group FA, with Group Model.

‡ the P-value for comparing Group FA, with Group FA + KO.

## 4 Discussion

In this study, we investigated the effects of FA on plaque vulnerability and AS progression, focusing on the IGF1/IGF1R signaling pathway and SMC differentiation. Consistent with previous studies (Kwon et al., 2010), our data showed that FA significantly reduces atherosclerotic plaque area and its vulnerability in ApoE^−/−^ mice. The key findings are as follows: (1) FA upregulates IGF1 expression in SMCs and activates the IGF1/IGF1R pathway in atherosclerotic plaques; (2) FA increases anti-inflammatory IL-4 expression and decreases pro-inflammatory IL-6 and MMP3 expression in an IGF1-dependent manner; (3) FA suppresses 45 kDa LAMP2 expression through IGF1 signaling; and (4) SMC-specific IGF1 deletion or IGF1R inhibition attenuates the protective effects of FA.

### 4.1 FA suppresses 45 kDa LAMP2 expression via IGF1 signaling

LAMP2 is a lysosomal membrane protein essential for autophagy and lysosomal biogenesis (Qiao et al., 2023). Its maturation requires N-linked glycosylation, which is critical for proper folding, stability, and lysosomal trafficking (Gao et al., 2022). Loss of glycosylated LAMP2 in VSMCs impairs autophagy and promotes abnormal phenotypic differentiation (Nguyen et al., 2018). Importantly, functional LAMP2 (120 kDa) is reduced in atherosclerotic plaques, whereas its upregulation enhances lipophagy and suppresses atherosclerosis progression (Zhang et al., 2021; Song et al., 2024; Huang et al., 2024).

Our finding that FA suppresses the bad-functional isoform through IGF1 signaling is particularly significant. The 45 kDa LAMP2 detected in this study likely represents a hypoglycosylated or immature isoform. While the precise identity of this isoform requires further characterization, its downregulation by FA-IGF1 signaling suggests a potential role in maintaining lysosomal function.

By reducing the expression of the hypoglycosylated variant, FA may effectively increase the proportion of functional, fully glycosylated LAMP2 available for lipophagy, thereby enhancing lipid droplet breakdown and cholesterol mobilization from foam cells. This interpretation is consistent with our findings that FA reduces lipid accumulation in SMC-derived foam cells and that these effects are reversed by IGF1R inhibition. The reciprocal regulation of IGF1 and 45 kDa LAMP2 establishes a previously unrecognized link between growth factor signaling and lysosomal quality control in SMC-derived foam cells.

### 4.2 FA rebalances inflammation and reduces MMP3 expression

Chronic inflammation is a hallmark of atherosclerosis. The balance between pro-inflammatory and anti-inflammatory cytokines critically regulates plaque cell behavior and phenotype, thereby influencing plaque progression and stability (Kong et al., 2022). Our finding that FA suppresses IL-6 while elevating IL-4 reveals a novel immunomodulatory mechanism underlying its anti-atherosclerotic effects. The complete abrogation of these effects in SMC-*Igf1*-KO mice demonstrates that SMC-derived IGF1 is indispensable for FA’s anti-inflammatory actions. Notably, these findings uncover an unexpected role for SMCs as active regulators of the plaque inflammatory milieu through IGF1 signaling, positioning FA as a modulator of this previously unrecognized SMC-IGF1-cytokine axis.

MMP3 degrades extracellular matrix components, promoting plaque instability and increasing the risk of acute coronary syndromes. Its activation is also linked to inflammatory pathways (Roa-Bruzon et al., 2025). Our observation that FA significantly suppresses MMP3 expression in an IGF1-dependent manner identifies a novel mechanism by which FA may promote plaque stabilization. By reducing MMP3-mediated matrix degradation, FA could help maintain fibrous cap integrity and prevent plaque rupture.

### 4.3 The dual role of IGF1/IGF1R signaling in atherosclerosis

The IGF1/IGF1R pathway plays a complex, context-dependent role in AS. As an essential growth factor, IGF1 regulates cell growth, differentiation, survival, and tissue repair (Higashi et al., 2016). The effects of IGF1/IGF1R in AS have been described as “double-edged” (Shai et al., 2010). On one hand, IGF1 may promote early neointimal hyperplasia by stimulating SMC growth and migration. On the other hand, IGF1 enhances plaque stability by increasing fibrous cap thickness and reducing inflammation. Our previous study demonstrated that reduced IGF1 levels are associated with plaque rupture, while elevated IGF1 levels are associated with plaque erosion (Liu et al., 2020).

Considering that FA upregulated IGF1 expression and activated the IGF1/IGF1R pathway in our study, we hypothesize that FA may facilitate the morphological transition of vulnerable plaques from a rupture-prone to an erosion-prone phenotype, which is associated with better clinical outcomes. Besides, our findings show that HFCD and IGF1 deficiency affect fasting blood glucose, but FA does not. This lack of systemic glucose modulation, together with the direct vascular action of FA observed *in vitro*, supports a locally mediated anti-atherosclerotic mechanism within the vascular wall rather than systemic metabolic regulation.

While we demonstrate that IGF1 negatively regulates LAMP2, the precise molecular mechanism (e.g., transcriptional vs. post-transcriptional) remains to be elucidated: i. The identity of the 45 kDa LAMP2 isoform is not clearly validated; its specificity and molecular nature require confirmation. Ii. Direct evidence linking LAMP2 suppression to enhanced Lipophagy and lipid clearance is lacking. The functional consequences of 45 kDa LAMP2 regulation on lipid phagocytosis and foam cell formation require further investigation. Iii. The translational relevance of our findings needs validation in human SMC-derived foam cell models. Future studies addressing these questions will further strengthen the mechanistic understanding of FA’s anti-atherosclerotic effects.

### 4.4 Limitations

Several limitations of this study should be acknowledged: i. The aortic tissue limited in quantity was utilized for detecting the expression of the target protein but not for isolating smooth muscle cells (SMCs) to confirm the deletion of the *Igf1* gene. Ii. Our study employed HFCD-fed mice to examine post-*Igf1* gene knockout expression levels to assess gene expression under pathological conditions. However, ideal conditions should be established under normal dietary regimens. Iii. Our study was conducted exclusively in male mice due to the Y chromosome-linked nature of the Cre transgene in our SMC-specific Igf1 knockout line. This limitation precludes conclusions regarding female subjects. Iv. Due to the high cost of mice used in this study and the prolonged breeding and modeling cycles, only six mice were employed per group which is less than most experiments.

## 5 Conclusion

In summary, this study demonstrates that FA reduces the vulnerability of atherosclerotic plaques and inhibits atherosclerosis progression. The mechanism may be achieved partially through SMC-derived IGF1 inhibiting 45 kDa-LAMP2, modulating the inflammatory balance (IL-6/IL-4) and regulating matrix metalloproteinase 3 (MMP3) expressions. The SMC-IGF1/LAMP2 axis may represent a potential therapeutic target for atherosclerosis. These findings provide new mechanistic insights into the anti-atherosclerotic effects of FA and support its potential utility as a therapeutic candidate for AS-related cardiovascular diseases.

## Data Availability

The original contributions presented in the study are included in the article/supplementary material, further inquiries can be directed to the corresponding authors.
